# Differences in Gene Expression Profiles between Early and Late Isolates in Monospecies *Achromobacter* Biofilm

**DOI:** 10.3390/pathogens6020020

**Published:** 2017-05-19

**Authors:** Signe M. Nielsen, Rikke L. Meyer, Niels Nørskov-Lauritsen

**Affiliations:** 1 Department of Clinical Medicine, Health, Aarhus University, DK-8200 Aarhus, Denmark; smni@clin.au.dk; 2 Department of Clinical Microbiology, Aarhus University Hospital, DK-8200 Aarhus, Denmark; 3 Interdisciplinary Nanoscience Center, Aarhus University, DK-8000 Aarhus, Denmark; rikke.meyer@inano.au.dk

**Keywords:** cystic fibrosis, adaptation, antimicrobial resistance, efflux pumps, sulfur metabolism, transcriptomics

## Abstract

Bacteria of genus *Achromobacter* are emerging pathogens in cystic fibrosis (CF) capable of biofilm formation and development of antimicrobial resistance. Evolutionary adaptions in the transition from primary to chronic infection were assessed by transcriptomic analysis of successive isolates of *Achromobacter xylosoxidans* from a single CF patient. Several efflux pump systems targeting antimicrobial agents were upregulated during the course of the disease, whereas all genes related to motility were downregulated. Genes annotated to subsystems of sulfur metabolism, protein metabolism and potassium metabolism exhibited the strongest upregulation. K+ channel genes were hyperexpressed, and a putative sulfite oxidase was more than 1500 times upregulated. The transcriptome patterns indicated a pivotal role of sulfur metabolism and electrical signalling in *Achromobacter* biofilms during late stage CF lung disease.

## 1. Introduction

*Achromobacter* species are emerging pathogens increasingly isolated from cystic fibrosis (CF) patients [1,2]. These bacteria are innately resistant to a wide spectrum of antimicrobial agents and have the potential to develop pan-resistance. In the CF lung, colonising bacteria are subjected to selective pressures arising from the host immune system, antimicrobial treatments, competition with co-infecting microorganisms and steep oxygen- and nutrient gradients within biofilms [3,4,5]. Colonising bacteria undergo an evolutionary adaptation in transition from primary to chronic infection. Acute virulence factors such as the type III secretion system, cell cytotoxicity, motility and adhesion mechanisms become unimportant, whereas genes encoding antimicrobial resistance, exopolysaccharide expression and alternative metabolic pathways are upregulated [6]. *Achromobacter* sp. have the ability to form biofilms [7,8,9], which is important for establishment and maintenance of persistent infections [10] including those occurring in CF. Gene expression profiles and antimicrobial susceptibility at biofilm stage differ from planktonic cells [11]. To clarify adaptive mechanisms of *Achromobacter* we quantitated biofilm stage gene expression of three *Achromobacter xylosoxidans* isolates cultured from a single CF patient during a time-span of seven years.

## 2. Results and Discussion

A patient affiliated with the CF centre at Aarhus University Hospital experienced a first-time detection of *A. xylosoxidans* at the end of 2007. Transition to chronic infection rapidly ensued; the strain was cultured in six of 10 sputum samples during 2008 and in eight of 10 samples during 2009. Three isolates were examined in this study, namely the first-time detected isolate (CF2-a designated “early”) and two isolates obtained approximately one and seven years later (CF2-b and CF2-d designated “intermediate” and “late”, respectively). Using the European Committee on Antimicrobial Susceptibility Testing (EUCAST) interpretative criteria for *Pseudomonas* spp. (http://www.eucast.org/clinical_breakpoints/), the primary isolate was susceptible to *Pseudomonas*-β-lactams and colistin, but resistant to fluoroquinolones and aminoglycosides (Table 1). The minimal inhibitory concentrations (MICs) of tigecycline and trimethoprim/sulfamethoxazole were low, but interpretative criteria for these agents are not established [12].

Only modest increases in MICs were detected for later isolates. The MICs of penicillin-class β-lactams (piperacillin and ticarcillin) were unaltered; the MIC of cephalosporin-class β-lactam ceftazidime doubled from 4 to 8 mg/L but did not transgress the susceptibility breakpoint; however, the MICs of several carbapenem-class β-lactams increased and reached the intermediate category for doripenem and meropenem. An explicit increase in colistin MIC was apparent, from 1 mg/mL in the early and the intermediate isolate (susceptible), to >8 mg/L in the late isolate (resistant). Colistin belongs to the class of polypeptide antibiotics known as polymyxins that binds to lipopolysaccharides and phospholipids in the outer cell membrane of Gram-negative bacteria, which leads to disruption of the outer cell membrane and bacterial death. Colistin is commonly used for inhalation therapy of CF patients and attains considerable concentrations in airway secretions [13,14]. 

To elucidate putative mechanisms involved in adaptation to the CF lung and development of antimicrobial resistance, successive isolates of the same strain were propagated in monospecies biofilm in vitro and subjected to transcriptomic analysis.

### 2.1. Gene Expression Profiles

Transcriptional levels of 5939 coding sequences annotated by Rapid Annotation using Subsystem Technology (RAST) [15] were compared between the first-time cultured isolate and after approximately one and seven years of colonisation. When significant differences in gene expression were observed, the largest difference was never observed in comparison with the intermediate isolate, indicating a progression of gene regulation from the early- to the late-isolate. The presented data therefore focus on the comparison between the early- and the late-isolate. Differential expression was calculated based on gene expression levels derived from an average of three replicates from individually cultured biofilms. A total of 247 genes were upregulated five-fold or more in the late isolate. Of these, 132 were hypothetical proteins or of unknown function. A total of 157 genes were downregulated five-fold or more in the late isolate; 62 were hypothetical proteins or of unknown function. Two hundred and ten up- or down-regulated genes of known or presumed function were distributed into 22 subsystem categories by RAST annotation (Table S1). Table 2 lists 10 selected subsystem categories encompassing 77 genes that exhibited a five-fold or larger difference in expression between the early- and the late-isolate when cultured in monospecies biofilm in vitro; 52 were upregulated and 25 were downregulated.

Ten genes belonging to the type III secretion system were downregulated eight- to 29-times in the late isolate (Table 2). The type III secretion system is considered part of an acute virulence mechanism, rendering bacteria capable of infecting host cells [6,16,17]. Reduced expression of the type III secretion system is described for chronic infection in CF with *Pseudomonas aeruginosa* [18,19,20]; our data suggests that type III secretion is important for primary infection with *A. xylosoxidans*, whereafter the mechanism is downregulated, possibly as a means of conserving energy. Furthermore, three genes involved in flagellar motility were downregulated more than fivefold in the late isolate; actually, all 22 genes related to motility were downregulated (two-fold or more, Table S1). Flagellar motility has proven important for adhesion and invasion of host cells during early (acute) infection, but is no longer crucial in established infections [21]. Alveolar macrophages and polymorphonuclear leukocytes are less capable of phagocytosing *Pseudomonas aeruginosa* with loss-of-function mutations in flagellar motility genes, hence downregulation of flagellar motility may enable immune system evasion [22].

Three genes involved in recombination and repair of DNA were downregulated in the late isolate, which could indicate an increased mutation frequency (Table 2). However, longitudinal analysis of isolates of the present strain did not reveal transformation into the hypermutator phenotype [23].

Eight genes associated with anaerobic respiration were significantly upregulated in the late isolate, whereas cytochrome *o* ubiquinol oxidase genes associated with aerobic respiration were downregulated (Table 2). *P. aeruginosa* can adapt to the oxygen-restricted conditions found in the lungs of patients with CF, where it can utilize nitrite as energy source under anoxic conditions and ferment amino acids in the absence of nitrite [24]. *Achromobacter* also has the capacity to utilize nitrite as terminal electron acceptor in the absence of oxygen [8]. The observed regulation of respiratory genes indicates that *A. xylosoxidans*, like *P. aeruginosa*, can adapt to the microaerobic and anaerobic conditions prevalent in late stage CF by regulation of metabolic pathways.

**Figure 1:**
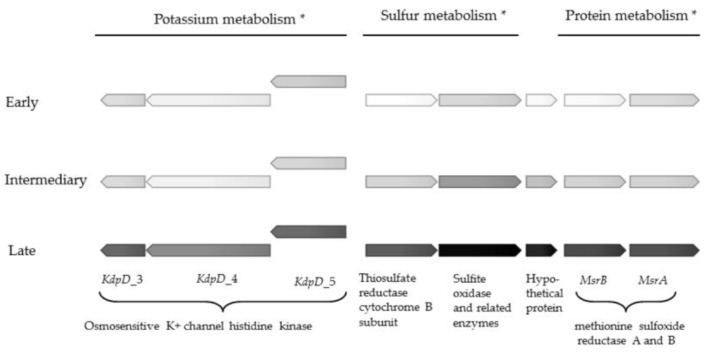
Gene expression in genes involved in protein, sulfur and potassium metabolism in early, intermediate and late isolate. Darker colour corresponds to higher gene expression, ranging from 16 to 38,628 reads, normalized to reads per kilobase per million mapped reads (RPKM), averaged from three replicates. Exact gene expression values are shown in Table S1. * Gene function according to Rapid Annotation using Subsystem Technology (RAST) annotation.

The most pronounced upregulation of genes of known function were observed in the subsystems categorised as sulfur metabolism, protein metabolism and potassium metabolism (Table 2). One gene encoding a putative sulfite oxidase was more than 1500 times upregulated, and attained a transcriptional level only surpassed by hypothetical protein_418 (Table S1). Methionine sulfoxide reductase genes *MsrA* and *MsrB* were also highly upregulated (756 and 201 times, respectively) (Table 2 and Figure 1). Sulfur metabolism has been connected to biofilm metabolism in *Staphylococcus aureus* [25], to production of an adhesin in *Escherichia coli* [26], and with iron acquisition in *P. aeruginosa* [27]. The function of sulfur metabolism in chronic *Achromobacter* infections remains to be elucidated, but the pivotal upregulation suggests a key adaptive role of sulfur metabolism in late stage CF. 

Four osmosensitive K+ channel histidine kinase genes were also massively upregulated (up to 128 times) in the late isolate (Figure 1 and Table 2). To our knowledge, electrical signalling has not been investigated in CF pathogens, but potassium ion channels can promote biofilm formation and electrical signalling in biofilms of the unrelated Gram-positive species *Bacillus subtilis* [28,29,30]. Interestingly, the electrically mediated attraction appears to be a generic mechanism that enables cross-species interactions, as *P. aeruginosa* also become attracted to the electrical signal released by the *B. subtilis* biofilm [31] Upregulation of the *KdpD* genes in late isolates of *A. xylosoxidans* suggests that these genes confer an evolutionary advantage in chronic colonisation of the CF lung, at least for this species.

### 2.2. Antimicrobial Resistance

The macrolide-specific efflux proteins MacA and MacB were 23.5 and 7.5 times upregulated, respectively, and the Resistance-nodulation-cell division (RND)-type multidrug resistance efflux pump genes *AxyA* and *OprM* (annotated in RAST as *CmeA* and *CmeC*) were upregulated 21.1 and 6.1 times, respectively (Table 2 and Table S1). Furthermore, a beta-lactamase gene was 23.3 times upregulated in the late isolate. The transcriptional repressor located upstream of a class D beta-lactamase gene (*bla*_OXA-114_) was deleted in the late isolate [32]; however *bla*_OXA-114_ has a narrow-spectrum hydrolysis profile with little effect on *Achromobacter*-active agents [33]. In contrast, inactivation of the RND-type efflux pump AxyAB-OprM decreases the MICs of cephalosporins (except cefepime), aztreonam, nalidixic acid, fluoroquinolones, and chloramphenicol [34]. Thus, hyperexpression of *AxyA* and *OprM* is likely involved in the decreased susceptibility to some carbapenems and fluouroqionolones observed with the late isolate (Table 1). 

Antimicrobial resistance is an acquired, permanent alteration of the bacterial genome, whereas antimicrobial tolerance is caused by a reversible, altered mode of growth within biofilms that has been linked to starvation [35]. Antimicrobial tolerance was assessed by quantitation of the minimal biofilm eradication concentration (MBEC). Antimicrobial tolerance increased over time for all four tested antimicrobials, particularly colistin, where the MBEC increased from 128 µg/mL to 2048 µg/mL (Table 3). MIC measurements have the advantage of being fast and easily automated. Although MBEC may reflect biofilm tolerance more closely than MIC, a recent review evaluating clinical outcomes of *P. aeruginosa* treatment found insufficient evidence of improved outcomes of treatment based on MBEC testing [36].

The very high levels of antimicrobial tolerance in *Achromobacter* biofilm highlight the need for novel treatment methods to combat chronic infections in CF.

We tested the general efflux pump inhibitor phenylalanine arginyl β-naphthylamide (PAβN) for effects on abiotic adherence and antimicrobial susceptibility. Biofilm formation assessed as adhesion to abiotic surface was not affected by addition of PAβN (Figure S1), in contrast to results obtained with several Gram-positive and Gram-negative bacteria [37,38,39]. PAβN showed little effect on MIC, while MBEC was reduced for colistin (Table 3); however, this effect could not be demonstrated for the late isolate that is characterised by hyperexperession of several efflux systems. The EmrAB efflux pump system of *Acinetobacter baumannii* contribute to colistin resistance [40]; for *P. aeruginosa*, the MexAB-OprM efflux pump system (with strong homology to AxyAB-OprM [34]) is necessary for development of colistin-tolerant subpopulation in biofilm [41]. Although only a modest influence of PAβN on antimicrobial susceptibility was detected, the prominent effects associated with inactivation of the *AxyAB*-*OprM* and *AxyXY*-*OprZ* operons of *Achromobacter* [34,35,42] call for investigation of a wider spectrum of efflux pump inhibitors. The introduction of efflux pump inhibitors into clinical practice has, however, proven difficult due to toxicity of the compounds, low selectivity and stability and the ability to affect human cells [43].

Important insights have been gained from studies of gene expression in biofilms using in vitro model systems; however, caution must be taken when extrapolating results obtained from in vitro studies to in vivo conditions. Laboratory experiments cannot imitate crucial factors such as the intricate interplay between the infecting microorganism and the host immune system, as well as the physical and chemical environment in the CF airways [44]. We used the composite and nutrient-rich Brain Heart Infusion (BHI) growth medium, which may have masked some differences in expression of genes related to nutrition and starvation that could have been revealed by use of synthetic cystic fibrosis sputum medium (SCFM) [45].

In conclusion, transcriptome analysis of successive isolates of *A. xylosoxidans* from a chronically infected CF patient revealed metabolic alterations partly reflecting similar modifications observed with other aerobic Gram-negative pathogens, notably *P. aeruginosa*. A key adaptive role for regulation of sulfur metabolism, and a prominent upregulation of K+ channel histidine kinase genes require further analysis. Multidrug efflux pumps constitute putative targets for abolition of the distressing tendency of this species to develop resistance to antimicrobial agents

## 3. Materials and Methods

### 3.1. Strains and Growth Conditions

Three consecutive isolates of the same clinical strain of *A. xylosoxidans* were cultured at 37 °C on 5% blood agar or in BHI media with shaking at 180 rpm. Clonal relationship of the infecting strain of *A. xylosoxidans* has been confirmed by pulsed field gel electrophoresis in a previous study investigating the early- and intermediate isolates, plus a third isolate not used in the present study (isolates CF2-a, CF2-b and CF2-c in [32]). Growth rates of planktonic cultures were determined by optical density measurements at 600 nm using a Multiskan™ GO Microplate Spectrophotometer (Thermo Fisher Scientific, Waltham, MA, USA) in kinetic mode. Biofilm formation was quantified using the crystal violet microtiter assay as previously described [9].

### 3.2. Preparation of Biofilms and RNA Extraction

Biofilms were prepared in triplicate. Each of these triplicates were prepared from three separate overnight cultures, inoculated from separate colonies, adjusted to an OD_600_ of 0.1 (corresponding to approximately 10^6^ cells/mL), and 1 mL from each were pooled and mixed. Biofilms were formed in six-well cell culture plates (TC Plate 6 Well, Sarstedt, Nümbrecht, Germany) in BHI media, and grown for three days at 37 °C. One mL media from each well was gently removed every 24 h and replaced with fresh media. The medium was carefully removed after 72 h, and biofilms were gently rinsed five times with PBS to remove planktonic bacteria. The attached biofilm was scraped off the bottom of the wells using a sterile inoculation loop. Approximately 500 µL biofilm biomass was transferred to an Eppendorf tube and treated with RNA Protect (RNeasy Protect Bacteria Mini Kit, Qiagen, Hilden, Germany) according to manufacturer’s instructions, except that treatment was carried out twice with an extended treatment time of 15 min. The RNA was extracted using the RNeasy Protect Bacteria Mini Kit (Qiagen, Hilden, Germany) according to the manufacturer’s protocol for enzymatic lysis and proteinase K digestion of bacteria, using an extended lysis time of 30 min. Contaminant DNA was removed using the Turbo DNA-*free*™ Kit (Thermo Fisher Scientific, Waltham, MA, USA). rRNA was removed with Ribo-Zero™ rRNA Removal Kit (Bacteria) (Illumina, San Diego, CA, USA). 

### 3.3. Sequencing and Transcriptomic Data Processing

Library preparation was carried out using ScriptSeq™ Complete Kit (Bacteria)—Low Input (Illumina, San Diego, CA, USA), and sequencing was performed on an Illumina NextSeq 500 platform to a sequence depth of ~50 million reads and a length of 150 nucleotides per read. The quality of the raw data output was assessed with FastQC version 0.11.3 [46], and CLC Genomics Workbench (Qiagen). Trimming was applied to remove adapters and low quality data using CLC Genomics Workbench (Qiagen, Hilden, Germany), and the quality of the trimmed contigs was re-assessed using FastQC. Sequence reads of each experiment were normalized by the method of reads per kilobase per million mapped reads (RPKM). The early isolate has previously been sequenced and annotated (isolate CF-2a, [32]) and was used for mapping the reads. Differences in gene expression were considered statistically significant for p-values below 0.01 as suggested (user manual, CLC Genomics Workbench). 

### 3.4. Antimicrobial Susceptibility Measurements

Minimal inhibitory concentration was determined for 21 antimicrobial agents using MIC plates for Gram-negative rods (GNX2F) incubated for 20 h at 37 °C and analysed by the Sensititre^®^ Windows Software SWIN^®^ (Termo Fischer Scientific, Waltham, MA, USA) according to the manufacturer’s recommendation. Minimal biofilm eradication concentration (MBEC) was determined for four antimicrobials as previously described [9], except that the effect of the efflux pump inhibitor phenylalanine arginyl β-naphthylamide (PAβN) (Sigma-Aldrich, St. Louis, MO, USA) on MBEC was measured by adding 100 µg/mL PAβN to each well. The effect on MIC was measured by adding 100 µg/mL PAβN to microtiter wells containing different concentrations of selected antimicrobial agents before inoculation and incubation for 20 h.

## Figures and Tables

**Table 1:** Antimicrobial susceptibility of successive *Achromobacter xylosoxidans* isolates.

Antibiotic (µg/mL)	CF2-a (Early)		CF2-b (Intermediate)		CF2-d (Late)	
	MIC	Categorisation *	MIC	Categorisation *	MIC	Categorisation *
Amikacin	>32	R	>32	R	>32	R
Aztreonam	>16	R	>16	R	>16	R
Cefepime	16	R	8	S	>16	R
Cefotaxime	32	NI	>32	NI	>32	NI
Ceftazidime	4	S	4	S	8	S
Ciprofloxacin	2	R	2	R	>2	R
Colistin	1	S	1	S	> 8	R
Doripenem	0.25	S	0.25	S	2	I
Doxycycline	8	NI	8	NI	8	NI
Ertapenem	≤0.25	NI	1	NI	>4	NI
Gentamicin	>8	R	>8	R	>8	R
Imipenem	≤1	S	2	S	≤1	S
Levofloxacin	2	R	2	R	> 8	R
Meropenem	≤1	S	≤1	S	4	I
Minocycline	≤2	NI	≤2	NI	4	NI
Piperacillin	8	S	8	S	8	S
Polymyxin B	1	NI	1	NI	4	NI
Ticarcillin/Clavulanic Acid	≤16	S	≤16	S	≤16	S
Tigecycline	≤0.25	NI	0.5	NI	0.5	NI
Tobramycin	> 8	R	> 8	R	> 8	R
Trimethoprim/ Sulfamethoxazole	≤0.5	NI	≤0.5	NI	≤0.5	NI

* Interpreted according to EUCAST susceptibility breakpoints for *Pseudomonas* species; S: Sensitive; I: Intermediate resistance; R: Resistant; NI: No Interpretation; MIC: minimal inhibitory concentration.

**Table 2:** Differentially expressed genes in early and late isolates of *A.* *xylosoxidans.*

Gene Function	Downregulated Genes in the Late Isolate	Upregulated Genes in the Late Isolate	Fold Change *
*Virulence*			
Arsenic resistance *ArsH*		1	8.2
Zinc resistance		2	14.1 to 20.5
Type III secretion system	10		−29.0 to −7.8
*Motility*			
Flagellar motility	3		−11.3 to −5.4
*Antimicrobial susceptibility*			
Beta-lactamase		1	23.3
Multidrug resistance efflux pumps		4	6.1 to 23.5
*Cell Wall and Capsule*			
Capsular and extracellular polysaccharides	2	2	−9.4 to 5.2
Lipopolysaccharide assembly *YrbC*		1	8.8
Bacterial peptidoglycan hydrolases		1	9.2
EPS biosynthesis *EpsF*		1	5.9
Capsular polysaccharide ABC transporter *KpsT*		1	6.6
*Respiration*			
Anaerobic respiratory reductases		2	6.1 to 9.0
Formate dehydrogenase		2	5.3 to 6.7
Soluble cytochromes		1	9.6
Fermentation		1	7.3
Nitrogen Metabolism		1	7.2
Dentrification		1	46.0
Cytochrome O ubiquinol oxidase subunit I–IV	4		−20.7 to −7.3
Succinate dehydrogenase	1		−9.4
*Stress response*			
Cold shock *CspA*/*CspG*		2	5.5 to 10.7
Detoxification		1	10.4
Osmotic stress		3	5.1 to 8.6
Oxidative stress		2	9.5 to 11.8
Heat shock	1		−5.4
*DNA metabolism*			
DNA recombination *RuvA*/*RuvC*	2		−6.5 to −5.9
DNA repair *RecO*	1		−6.7
*Sulfur metabolism*			
Inorganic sulfur assimilation		4	10.1
Organic sulfur assimilation	1	6	−5.7 to 22.7
Sulfur metabolism		2	313.8; 1516.2
*Protein metabolism*			
Protein degradation		3	7.9 to 24.7
Protein biosynthesis		1	6.3
Protein processing and modification *MsrA* and *MrsB*		2	201.2 to 756.1
*Potassium metabolism*			
Osmosensitive K+ channel histidine kinase *KdpD* II-V		4	8.7 to 128.0

* Late isolate compared with early isolate.

**Table 3:** Minimal biofilm eradication concentration (MBEC) of selected antimicrobials with and without addition of efflux pump inhibitor PaβN.

Antibiotic (µg/mL)	CF2-a (Early)		CF2-b (Intermediate)		CF2-d (Late)	
	MIC	MBEC	MIC	MBEC	MIC	MBEC
Colistin	1	128	1	128	>8	2048
+PAβN (100 µg/mL)	1	≤32	1	64	>8	2048
Levofloxacin	2	128	2	128	>8	256
+PAβN (100 µg/mL)	2	128	2	128	4	256
Tobramycin	>8	512	>8	>1024	>8	>1024
+PAβN (100 µg/mL)	>8	512	>8	>1024	>8	>1024
Piperacillin	8	2048	8	2048	8	>2048
+PAβN (100 µg/mL)	8	2048	8	2048	8	>2048

## Supplementary Materials

The following are available online at www.mdpi.com/2076-0817/6/2/20/s1, Figure S1: Biofilm formation in the presence and absence of efflux pump inhibitor PaβN; Figure S2: Growth curves of early, intermediary and late isolates of *A. xylosoxidans*; Table S1: Complete list of differentially expressed genes including gene expression values.
