# Supplementary material for: Differences in Gene Expression Profiles between Early and Late Isolates in Monospecies Achromobacter Biofilm
Source: Pathogens. 2017 May 19;6(2):20. doi: 10.3390/pathogens6020020 (PMC5488654; doi:10.3390/pathogens6020020)
Supplement: Supplementary file 1 [file pathogens-06-00020-s001.zip › Figure S1.pdf]

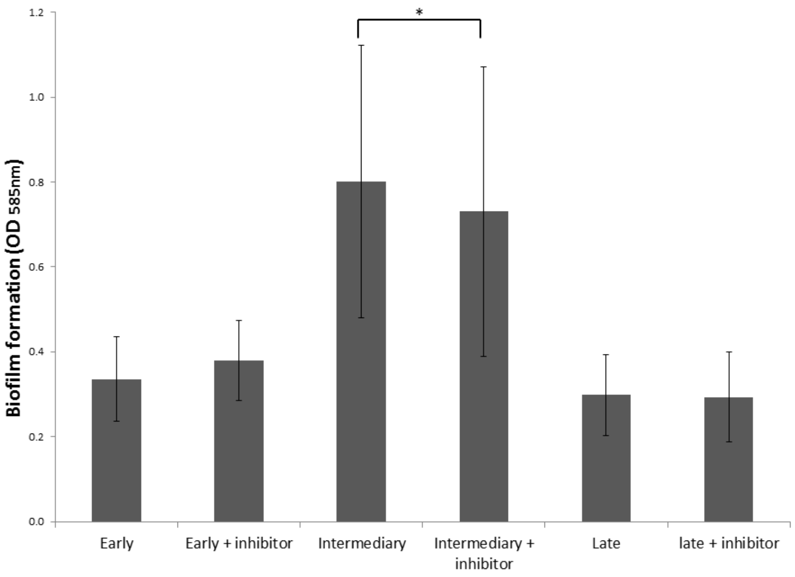

Figure S1. Biofilm formation in the presence and absence of efflux pump inhibitor PaβN

* Statistically significant difference, $p = 0.0004$

Overnight cultures were grown in BHI and adjusted to OD600 = 0.1 with fresh BHI, and 160 μL per well was added to 96 well micro titer plates. 100 μg/mL of the efflux pump inhibitor phenylalanine arginyl β-naphthylamide (PAβN) (Sigma-Aldrich) was added to the wells before peg-lids were inserted. After 24 h incubation at 37 °C, biofilm formation in the presence of each enzyme was quantified by crystal violet staining as described in Nielsen *et. al.* [9], and compared to controls with no addition of PAβN. The experiments were carried out three times with eight replicates in each experiment.
