# Supplementary material for: Differences in Gene Expression Profiles between Early and Late Isolates in Monospecies Achromobacter Biofilm
Source: Pathogens. 2017 May 19;6(2):20. doi: 10.3390/pathogens6020020 (PMC5488654; doi:10.3390/pathogens6020020)
Supplement: Supplementary file 1 [file pathogens-06-00020-s001.zip › Figure S2.pdf]

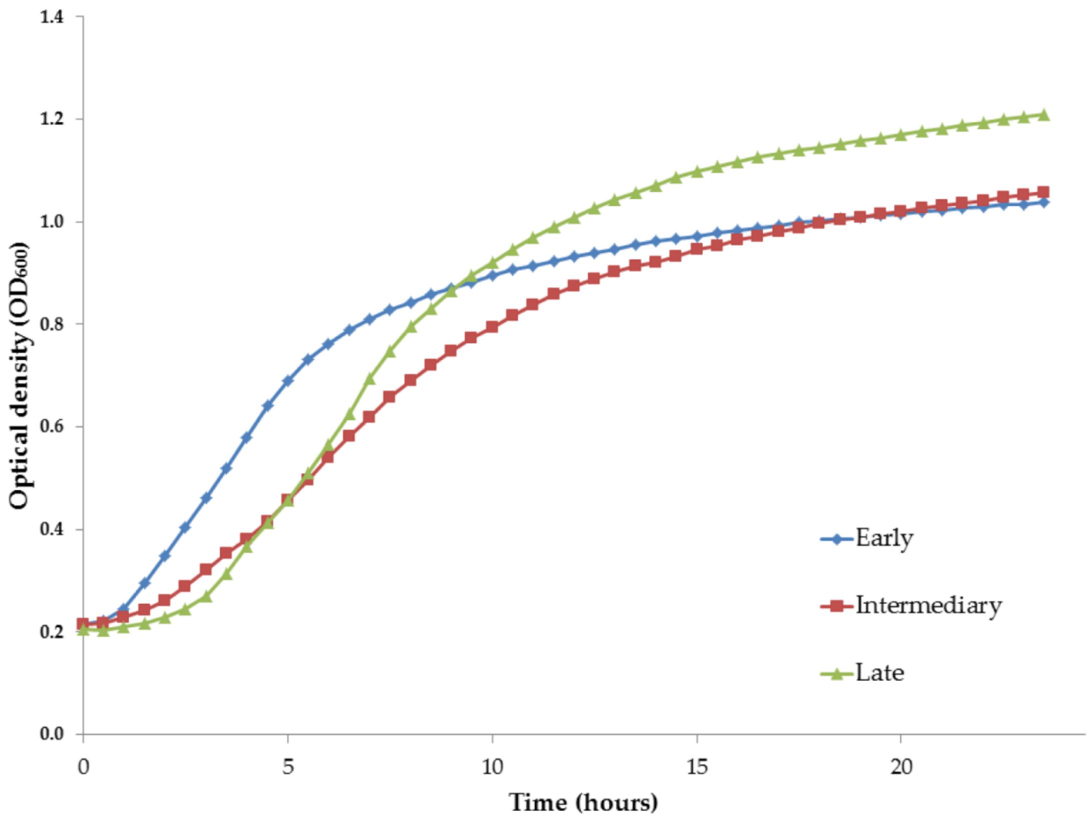

Figure S2. Growth curves of early, intermediary and late isolates of *A. xylosoxidans*

Growth rates of the early, intermediary and late isolates were determined by optical density measurements at 600 nm in 96 well plates with 200 µL per well. Isolates were cultured in BHI media at 37°C with continuous shaking. Measurements were taken every 30 min using a Multiskan™ GO Microplate Spectrophotometer (Thermo Fisher Scientific) in kinetic mode.
